# Metabolite Genome-Wide Association Study for Indoleamine 2,3-Dioxygenase Activity Associated with Chronic Kidney Disease

**DOI:** 10.3390/genes12121905

**Published:** 2021-11-27

**Authors:** Hye-Rim Kim, Hyun-Seok Jin, Yong-Bin Eom

**Affiliations:** 1 Department of Medical Sciences, Graduate School, Soonchunhyang University, Asan 31538, Chungnam, Korea; goa6471@naver.com; 2 Department of Biomedical Laboratory Science, College of Life and Health Sciences, Hoseo University, Asan 31499, Chungnam, Korea; jinhs@hoseo.edu; 3 Department of Biomedical Laboratory Science, College of Medical Sciences, Soonchunhyang University, Asan 31538, Chungnam, Korea

**Keywords:** indoleamine 2,3-dioxygenase (IDO), estimated glomerular filtration rate (eGFR), chronic kidney disease (CKD), single-nucleotide polymorphism (SNP), metabolite genome-wide association study (mGWAS)

## Abstract

Chronic kidney disease (CKD) causes progressive damage to kidney function with increased inflammation. This process contributes to complex amino acid changes. Indoleamine 2,3-dioxygenase (IDO) has been proposed as a new biomarker of CKD in previous studies. In our research, we performed a metabolite genome-wide association study (mGWAS) to identify common and rare variants associated with IDO activity in a Korean population. In addition, single-nucleotide polymorphisms (SNPs) selected through mGWAS were further analyzed for associations with the estimated glomerular filtration rate (eGFR) and CKD. A total of seven rare variants achieved the genome-wide significance threshold (*p* < 1 × 10^−8^). Among them, four genes (*TNFRSF19*, *LOC105377444*, *LOC101928535*, and *FSTL5*) associated with IDO activity showed statistically significant associations with eGFR and CKD. Most of these rare variants appeared specifically in an Asian geographic region. Furthermore, 15 common variants associated with IDO activity were detected in this study and five novel genes (*RSU1*, *PDGFD*, *SNX25*, *LOC107984031*, and *UBASH3B*) associated with CKD and eGFR were identified. This study discovered several loci for IDO activity via mGWAS and provided insight into the underlying mechanisms of CKD through association analysis with CKD. To the best of our knowledge, this is the first study to suggest a genetic link between IDO activity and CKD through comparative and integrated analysis.

## 1. Introduction

Chronic kidney disease (CKD) is caused by several factors, including diabetes, high blood pressure, and glomerulonephritis [1]. CKD can lead to end-stage renal failure due to the gradual loss of kidney function and fibrosis caused by inflammation [2,3]. The Centers for Disease Control and Prevention (CDC) have reported that 90% of adults with CKD are unaware that they have CKD [4]. The early stages of CKD have few signs or symptoms. However, the progression of CKD increases cardiovascular morbidity and mortality, making treatment difficult [5]. Therefore, the management of patients with CKD should focus on delaying disease progression by identifying risk factors through early diagnosis [6].

Metabolomics is a field of interest in nephrology because many metabolites, which are small molecules, are freely filtered by the kidneys [7]. In addition, metabolites that play important roles in numerous biological pathways are also known as potential biomarkers of several diseases, including CKD [8,9,10]. Currently, the estimated glomerular filtration rate (eGFR) calculated by creatinine is mainly used for the diagnosis of kidney function [11]. However, previous studies have reported that it is difficult to use creatinine to detect early kidney damage [12,13]. For this reason, new biomarkers that can identify kidney damage at an early stage are needed.

Indoleamine 2,3-dioxygenase (IDO) is an enzyme involved in the metabolism of tryptophan belonging to the kynurenine pathway. It is induced by pro-inflammatory cytokines [14]. It is evaluated as the kynurenine to tryptophan ratio (K/T ratio) [15]. IDO is also known to play an important role in immunological processes such as infection, autoimmunity, and chronic inflammation [16]. Moreover, Mohib et al. [17] have reported that IDO induced by pro-inflammatory cytokines plays an apoptotic role in renal tubular epithelial cells. Since the kidneys are involved in the clearance of tryptophan metabolites, their impairment is associated with increased tryptophan metabolism and IDO activity [18,19,20]. Interestingly, one study has reported that CKD-induced IDO activity is correlated with CKD severity and major inflammatory markers such as high-sensitivity C-reactive protein (hs-CRP) and soluble TNF-receptor-1 (sTNFR-I) [15]. In addition, Lee et al. [21] have demonstrated an association between IDO activity and CKD in Koreans.

Previous studies examining the genetic influence of CKD patients have estimated the heritability of CKD to be 30–75% [22,23]. In this regard, several studies have identified many genetic loci associated with CKD through genome-wide association analysis [24,25]. Although genome-wide association study (GWAS) may indicate risk for disease, it does not clearly explain the underlying cause. Thus, other research groups have performed an integrated analysis of GWAS with other fields such as metabolomics, proteomics, and transcriptomics to overcome the limitations of GWAS [26,27]. To the best of our knowledge, metabolite GWAS (mGWAS) for IDO activity associated with CKD has not been reported yet. Therefore, this study performed mGWAS to identify common and rare variants associated with IDO activity using the Korean Association REsource (KARE) cohort. To find a biological link between genetic loci and CKD, we further analyzed associations of single-nucleotide polymorphisms (SNPs) related to IDO activity with CKD and eGFR.

## 2. Materials and Methods

### 2.1. Ethics Statement

This study was approved by the Institutional Review Board (IRB) of the Korea Disease Control and Prevention Agency (KDCA, KBN-2021-003, 26 January 2021) and Soonchunhyang University (202012-BR-086-01, 15 December 2020). Written informed consent was obtained from all participants.

### 2.2. Study Participants

This study used the Korean Association REsource (KARE) cohort as part of the Korean Genome and Epidemiology Study (KoGES). The KARE cohort is a community-based cohort in Ansung (rural) and Ansan (urban) areas. Follow-up surveys were conducted every two years from 2001 to 2014. Among them, secondary follow-up data (2005–2006) with metabolite information were used as baseline data. A total of 2579 participants aged 43–74 years were included in the present study. The KARE cohort has been previously described in detail [28]. According to the criteria of the Kidney Disease Improving Global Outcome (KDIGO), participants were classified into CKD (cases, *n* = 264) and non-CKD (controls, *n* = 1550) groups based on eGFR. CKD was defined as eGFR levels below 60 mL/min/1.72 m^2^. Non-CKD was defined as an eGFR of more than 60 mL/min/1.72 m^2^. Participants with a history of hypertension and diabetes as risk factors for kidney disease and those who were taking medications related to these diseases were excluded from the control group.

### 2.3. General Characteristics

Anthropometric and biochemical information of participants was obtained from the KARE database. General characteristics of participants are listed in Table 1. Height (m) and weight (kg) were measured using an automated measuring instrument (Dong Sahn Jenix Co., Seoul, Korea) three times to obtain average values. Body mass index (BMI; kg/m^2^) was calculated as weight/squared height. Blood pressure was measured with a mercury sphygmomanometer (Baumanometer; W.A. Baum, Copiague, NY, USA). Biochemical parameters such as serum creatinine and blood urea nitrogen (BUN) levels were analyzed through blood sampling. Creatinine levels were assessed with the Jaffe method using an automatic analyzer (Hitachi, Tokyo, Japan). eGFR was calculated using the CKD-Epidemiology Collaboration formula as follows: GFR = 141 × min (creatinine/κ, 1)^α^ × max (creatinine/κ, 1)^−1.209^ × 0.993^age^ × gender (for women, gender = 1.018; α = −0.329; κ = 0.7; for men, gender = 1; α = −0.411; κ = 0.9).

### 2.4. Metabolite Measurements

To quantify kynurenine and tryptophan, serum samples collected from 2579 participants were analyzed using an AbsoluteIDQ p180 kit (BIOCRATES Life Science, Innsbruck, Austria) according to the manufacturer’s instructions. Liquid chromatography/tandem mass spectrometry (LC–MS/MS) was conducted using an API 4000 QTRAP system (Applied Biosystems, Foster City, CA, USA) equipped with an Agilent 1200 HPLC system (Agilent Technologies, Santa Clara, CA, USA) to measure metabolites. The quality control (QC) process for analyzed metabolites has been described in detail elsewhere [29]. Briefly, both kynurenine and tryptophan used in this study met the following criteria: the coefficient of variance for each metabolite in the reference standards < 25%, 50% of the analyzed metabolite concentrations in the reference standards > limit of detection, and 50% of the analyzed metabolite concentrations in the experimental samples > limit of detection. Pooled human normal serums were used as reference standards. IDO activity was estimated as the ratio of kynurenine to tryptophan.

### 2.5. Genotyping and Imputation

Genotyping of the KARE dataset was performed using an Affymetrix Genome-Wide Human SNP array 5.0 (Affymetrix, Santa Clara, CA, USA). QC criteria of samples and variants have been previously described [30]. Briefly, samples with low call rates (<96%), DNA contamination, gender inconsistency, and serious concomitant illnesses were excluded. Exclusion criteria for variants were: Hardy–Weinberg equilibrium (HWE) *p*-values < 1 × 10^−6^, missing call rates > 5%, and minor allele frequency (MAF) < 0.01. After the QC process, imputation analysis of genetic variants was performed using an IMPUTE2 program with 1000 Genomes Phase I data as a reference panel [31]. A total of 6,461,358 SNPs were included in this study. Locations of variants were assigned using National Center for Biotechnology Information (NCBI) Human Genome Build 37 (hg19).

### 2.6. Statistical Analysis

All statistical analyses were conducted with PLINK version 1.90 β (https://www.cog-genomics.org/plink2 (accessed on 26 July 2021)) [32]. Linear regression was used to assess associations of variants with IDO and eGFR. A case–control study was performed using logistic regression analysis. All regression analyses were based on an additive model and adjusted for age, gender, area, BMI, hemoglobin A1C (HbA1c), drinking, smoking, systolic blood pressure (SBP), and hs-CRP. The cutoff *p*-value was *p* < 5 × 10^−8^ for rare variants and *p* < 1 × 10^−5^ for common variants. Statistical significance between two groups was confirmed via Student’s *t*-test. After performing mGWAS for IDO activity, linkage disequilibrium (LD) among variants was considered through clumping analysis. Variants were clumped through the following criteria: significance threshold of *p* < 0.05, LD threshold < 0.5, and physical distance threshold < 1000 kb. The variant with the lowest *p*-value among clumped variants was selected as the index variant. Manhattan plot and LD block were drawn using the Haploview version 4.1 program (Whitehead Institute for Biomedical Research, Cambridge, MA, USA). Geographical distribution maps for variants were generated based on the 1000 genome database via the Geography of Genetic Variants (GGV) browser (http://popgen.uchicago.edu/ggv/ (accessed on 20 August 2021)). LocusZoom browser (http://locuszoom.org/ (accessed on 16 September 2021)) was used to draw regional plots. Kyoto Encyclopedia of Genes and Genomes (KEGG) pathway database (https://www.genome.jp/kegg/ (accessed on 2 September 2021)) was used to investigate biological processes involved in IDO activity and CKD.

## 3. Results

### 3.1. Participant Characteristics

The baseline characteristics of the study population are shown in Table 1.

A total of 2579 participants with metabolite and genotype data were included in this study. To investigate the genetic association of IDO activity and CKD, participants were divided into cases with CKD (*n* = 264) and controls (*n* = 1550). The mean age of all participants was 57.10 ± 9.05 years, with the case group being older (mean age: 65.72 ± 6.53 years) than the control group (mean age: 54.98 ± 8.64 years). In addition, values of kidney-related traits such as eGFR, creatinine, and BUN showed significant differences (*p* < 0.001) between cases and controls.

### 3.2. Associations between Common Variants and IDO Activity Related to CKD

**Figure 1:**
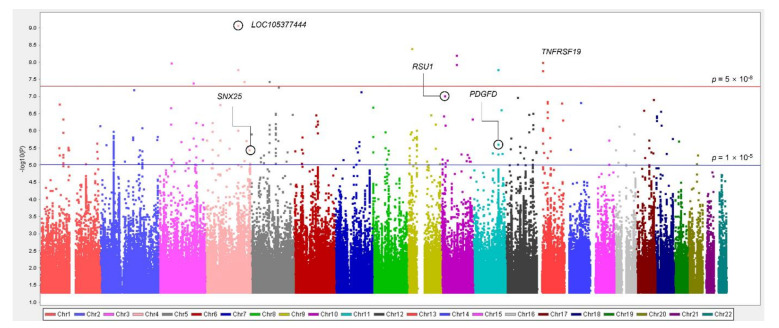
Manhattan plot of results from metabolite GWAS analysis of IDO activity. Red line indicates genome-wide significance threshold at *p* < 1 × 10^−8^. Blue line represents significance threshold at *p* < 1 × 10^−5^. Vertical axis indicates −log_10_ *p* values from linear regression adjusted for age, area, gender, BMI, drinking, smoking, SBP, hs-CRP, and HbA1c. Horizontal axis shows chromosomal positions. The Manhattan plot was generated with the Haploview program.

This study performed mGWAS of IDO activity in the KARE dataset and summarized the association between SNPs and IDO activity through a Manhattan plot (Figure 1).

As a result, 15 SNPs with MAF > 0.05 passed the significant threshold of 1 × 10^−5^ (Table 2). Of these, the strongest association with IDO activity was observed for rs59178336 in the *RSU1* (Ras Suppressor Protein 1) gene located on chromosome 10. We further studied associations of variants related to IDO activity with eGFR and CKD. We found that rs59178336, a variant with the lowest *p*-value for IDO activity, showed a significant association with CKD (*p* < 0.05). Minor allele carriers of rs59178336 significantly increased both the IDO level (*β* = 0.26, *p* = 9.41 × 10^−8^) and CKD risk (OR = 1.47, 95% CI: 1.02–2.14, *p* = 0.041). Although rs12226572 in the *UBASH3B* (Ubiquitin Associated and SH3 Domain Containing B) gene was significantly associated with CKD (*p* < 0.025), it did not exhibit a strong LD with surrounding SNPs at the 11q24.1 locus. Three SNPs (rs2513735, *p* = 0.012; rs78259836, *p* = 0.024; rs7237751, *p* = 0.043) in the *PDGFD* (Platelet-Derived Growth Factor D), *SNX25* (Sorting Nexin 25), and *LOC107984031* genes were also identified as significant variants for eGFR. Regional plots around the *RSU1*, *PDGFD*, *SNX25*, and *LOC107984031* genes revealed several SNPs in the LD, with top SNPs involved in IDO activity (Figure S1). On chromosome 11, rs2513735 near the *PDGFD* gene showed the most significant result for eGFR (*β* = −1.45, *p* = 0.012) among common variants. Additionally, the mitogen-activated protein kinase (MAPK) pathway known to regulate various cellular processes was identified through KEGG pathway analysis of the *PDGFD* gene (Figure S2).

### 3.3. Associations between Rare Variants and IDO Activity Related to CKD

**Figure 2:**
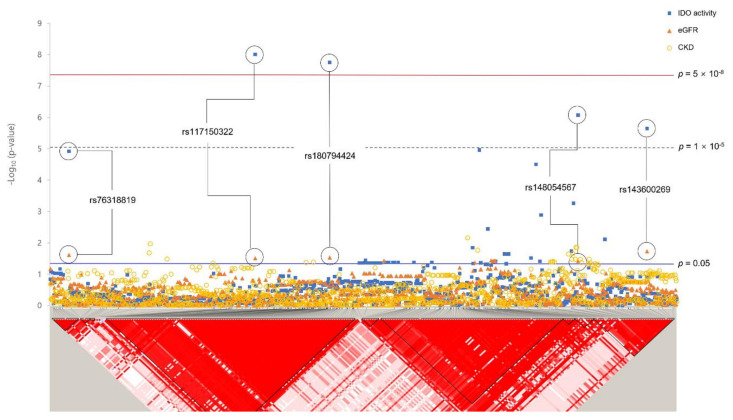
Plot of *p*-values of studied variants within *TNFRSF19* ± 100 kb regions. Results of association analysis between variants and IDO activity, eGFR, and CKD are plotted. Standard significant *p*-value threshold (*p* = 0.05) and GWAS *p*-value threshold (*p* = 5 × 10^−8^) are indicated by blue and red lines, respectively. The bottom panel shows a Haploview of LD (*r^2^*) based on genotyping data from KARE data. It was generated using the Haploview program.

A total of seven SNPs reached the GWAS threshold (*p* < 5 × 10^−8^), all of which were rare, with MAF < 0.05 (Table 3). Among these, rs182145739, located in the *LOC105377444* gene on chromosome 4, reached significance for both IDO activity (*p* < 5 × 10^−8^) and CKD (*p* < 0.05). The minor allele T of rs182145739 was positively associated with the IDO level (*β* = 0.82, *p* = 8.46 × 10^−10^). In addition, CKD risk was increased (OR = 2.63, 95% CI 1.15–6.03, *p* = 0.022) in minor allele carriers. Association analysis for rare variants with eGFR showed that three loci (near *TNFRSF19*, TNF Receptor Superfamily Member 19; *LOC101928535*, *FSTL5*, Follistatin Like 5) passed a significant threshold of 0.05. Figure 2 shows regional association plots for IDO activity, eGFR, and CKD of *TNFRSF19* ± 100 kb (13q12.12) with the LD block structure. Five SNPs (rs76318819, rs117150322, rs180794424, rs148054567, and rs143600269) plotted were significantly associated with both IDO activity and eGFR. Among them, rs117150322 and rs180794424, rs148054567 and rs143600269 were included in the same LD block. Rare variants passing the significant threshold of 1 × 10^−5^ are shown in Table S1.

### 3.4. Geographical Distribution of Rare Variants

**Figure 3:**
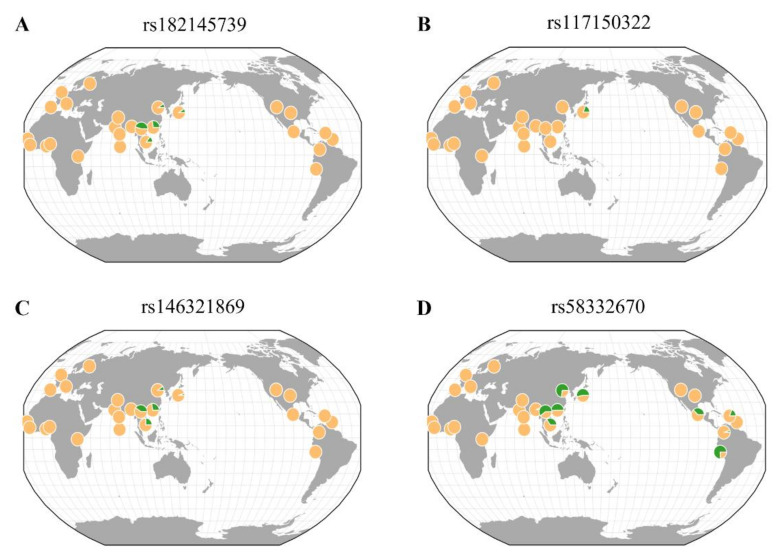
Geographical distributions of rare variants associated with eGFR and CKD. Each pie chart shows a minor allele frequency based on 1000 genomes (hg 19). The frequency scale of the green pie is the proportion out of 0.1. (**A**) rs182145739, (**B**) rs117150322, (**C**) rs146321869, (**D**) rs58332670.

This study further analyzed the geographic distributions of rare variants associated with eGFR and CKD, as well as IDO activity using the GGV browser (Figure 3). Rare variants (rs182145739, rs117150322, and rs146321869) were mostly seen in East Asia. For rs58332670, it was found in East Asia and America. In particular, rs117150322, located near the *TNFRSF19* gene, was detected only in a Japanese population (MAF = 0.019). The MAF of rs117150322 in Korea was 0.009, which was lower than that in Japan.

## 4. Discussion

Several studies have performed metabolite profiling to evaluate physiological pathways for CKD [15,33,34,35]. Interestingly, they have identified an association between CKD and the kynurenine pathway. According to another study, CKD is strongly associated with IDO activity, which degrades tryptophan to kynurenine in the kynurenine pathway [36]. Furthermore, IDO activity was positively correlated with CKD (OR = 12.65, 95% CI: 6.55–24.44) in a Korean population [21]. All of these studies were conducted during epidemiological investigations. Although CKD is a complex disease with high heritability, studies that perform a genetic analysis for the association between IDO activity and CKD have not been reported yet. Therefore, we performed mGWAS to identify genetic variants and potential loci affecting IDO activity associated with CKD in Koreans.

Our results revealed that 15 common variants had significant associations with IDO activity (*p* < 1 × 10^−5^) (Table 2) and that seven rare variants reached the GWAS threshold for IDO activity (Table 3). Additionally, SNPs related to IDO activity were analyzed for eGFR and CKD. Among genetic signals associated with eGFR and CKD, common variants were found at the *RSU1*, *PDGFD*, *SNX25*, *LOC107984031*, and *UBASH3B* genes and rare variants were identified at the *LOC105377444*, *TNFRSF19*, *LOC101928535*, *FSTL5* genes. We focused on the *RSU1*, *PDGFD*, *SNX25*, and *TNFRSF19* genes because evidence showing that other genes were associated with CKD was insufficient.

In the case of the *RSU1*, *PDGFD*, and *TNFRSF19* genes, they were associated with the MAPK pathway [37,38,39]. The *SNX25* gene was related to dopamine receptors. MAPK pathways include extracellular signal-regulated kinase (ERK), c-Jun N-terminal kinase (JNK), and p38 mitogen-activated protein kinase (p38MAPK) [40]. Although the MAPK pathway is generally known to regulate proliferation, many studies have suggested that it is an intracellular signaling pathway underlying kidney development [40,41,42]. Moreover, Fujigaki et al. reported that IDO activity is related to the MAPK pathways [43]. Dopamine receptors exist as D_1_-like (D_1_R and D_5_R) and D_2_-like (D_2_R, D_3_R, and D_4_R) subtypes according to their structure and pharmacology [44]. Among them, D_1_R is widely expressed in the kidney. It plays a central role in regulating blood pressure and sodium balance [45,46].

A recent study has reported that RSU1 is a critical mediator in downregulating ERK signaling through extracellular matrix (ECM) detachment [37]. The ERK pathway responsible for basic cellular processes is the most important signaling cascade of MAPK pathways [47]. It has also been reported that reduced ERK activity can improve antioxidant effects and kidney function [48]. In the present study, our results showed that, among common variants, rs59178336, located in the intron of the *RSU1* gene, was significantly associated with IDO activity and CKD. Interestingly, Reznichenko et al. [49] have confirmed that the *CUBN* gene, located close to the *RSU1* gene, is associated with end-stage renal disease. Therefore, the *RSU1* gene might be associated with kidney disease.

Our results also identified associations of regions near the *PDGFD* gene involved in ERK signaling with IDO activity and eGFR (Table 2, Figure S2) [38]. Similar to our results, a previous study has reported that a genetic variant (rs7103465) in the *PDGFD* gene is associated with the ratio of urine albumin to creatinine (*p* = 3.7 × 10^−7^) in Latin Americans [50]. Moreover, it has been reported that PDGFD is overexpressed in hepatic and renal fibrosis [51,52]. Charni et al. demonstrated that PDGFD is regulated by TGF-β, which activates MAPK pathways such as ERK, JNK, and p38MAPK [53].

Furthermore, it has been reported that SNX25 overexpression is associated with increased expression levels and signaling of D_1_R [54]. Another study has indicated that SNX5 depletion can result in hypertension in normotensive mice [55]. Therefore, the *SNX25* gene encoding protein SNX25 might be associated with hypertension, a risk factor for kidney disease. In our data, minor carriers of rs78259836 belonging to the *SNX25* gene increased IDO activity but decreased eGFR (Table 2).

Previous studies have reported that an understanding of progenitor cells involved in kidney damage and repair can provide insight into renal pathology and identify novel therapeutic targets [56,57]. Schutgens et al. [58] have demonstrated that *TNFRSF19* is a marker gene for epithelial progenitor cells that contributes to adult kidney development in vivo. Moreover, previous studies have reported that overexpression of the *TNFRSF19* gene can activate the JNK pathway and that its activation causes damage and fibrosis in the human kidney [39,59]. The JNK pathway involved in inflammation has also been reported as a mechanism regulating IDO [60,61]. Through mGWAS analysis, we discovered several SNPs located near the *TNFRSF19* gene associated with IDO activity (Table 3, Figure 2). The results of our study suggest that the *TNFRSF19* gene might regulate CKD by inducing IDO through JNK, which belongs to the MAPK family.

In summary, this study performed an mGWAS for IDO activity obtained from 2579 participants in the KARE cohort. A total of 22 novel SNPs (15 common and 7 rare variants) were found and further analyzed for genetic associations with eGFR and CKD. For variants selected based on metabolites, we investigated their associations with CKD compared to other study groups. As a result, four genes (*RSU1*, *PDGFD*, *SNX25*, and *TNFRSF19*) were associated with CKD by regulating IDO activity. In particular, our data highlight that the *RSU1* and *PDGFD* genes are potential mediators of CKD associated with ERK, which belongs to the MAPK family. The results of this study also suggest that rare variants of the *TNFRSF19* gene are associated with CKD, specifically in Asians, through the JNK pathway. The gene–metabolite associations identified in our study provide insight into the underlying mechanisms for CKD. However, functional analyses of mRNA expression levels and proteins should be performed to validate these findings. In addition, modern technological advances have made metabolite analysis possible, but the number of samples is still limited. Therefore, replication studies in other cohorts are needed to confirm the accuracy of this study.

## Figures and Tables

**Table 1:** Characteristics of participants in the Korean population.

Characteristics	Quantitative Trait Analysis	Case–Control Analysis for CKD		
		Controls	Cases	*p*-Value *
Number of participants	2579	1550	264	
Gender [men (%)]	1218 (47.23)	789 (50.90)	81 (30.68)	<0.001
Age (M years ± SD)	57.10 ± 9.05	54.98 ± 8.64	65.72 ± 6.53	<0.001
Height (M cm ± SD)	159.55 ± 9.16	160.55 ± 8.98	155.42 ± 8.28	<0.001
Weight (M kg ± SD)	62.63 ± 10.36	62.30 ± 10.41	60.88 ± 9.53	0.042
BMI (M kg/m^2^ ± SD)	24.56 ± 3.23	24.11 ± 3.09	25.20 ± 3.47	<0.001
eGFR (mL/min/1.73 m^2^)	75.58 ± 11.92	78.68 ± 9.69	55.24 ± 9.21	<0.001
Creatinine (mg/dL)	0.98 ± 0.20	0.96 ± 0.14	1.18 ± 0.42	<0.001
BUN (mg/dL)	15.69 ± 4.26	15.33 ± 3.92	17.91 ± 5.45	<0.001

BMI, body mass index; eGFR, estimated glomerular filtration rate; BUN, blood urea nitrogen; CKD, chronic kidney disease; M, mean value; SD, standard deviation. *, Significant differences in characteristics between cases and controls were determined with Student’s *t*-test.

**Table 2:** Common variants associated with indoleamine 2,3-dioxygenase activity in Koreans.

No.	SNP	Nearest Gene	Chromosome Position	Minor Allele	MAF	Function	IDO Activity		eGFR		CKD	
							*β* ± S.E.	*p*-Value	*β* ± S.E	*p*-Value	OR (95% CI)	*p*-Value
1	rs59178336	***RSU1***	10:16822091	C	0.095	Intron	0.26 ± 0.049	9.41 × 10^−8^	−0.59 ± 0.50	0.235	1.47 (1.02–2.14)	**0.041**
2	rs10469937	*CCDC85A*	2:56629317	C	0.435	-	−0.14 ± 0.028	1.03 × 10^−6^	0.35 ± 0.29	0.228	0.91 (0.73–1.14)	0.407
3	rs7588698	*HDAC4*	2:240041896	A	0.055	Intron	0.30 ± 0.062	1.42 × 10^−6^	−0.78 ± 0.64	0.225	1.43 (0.88–2.32)	0.145
4	rs2513735	***PDGFD***	11:104081184	T	0.065	-	0.27 ± 0.057	2.39 × 10^−6^	−1.45 ± 0.58	**0.012**	1.08 (0.70–1.67)	0.730
5	rs6730950	*RTN4*	2:55386276	C	0.149	-	0.19 ± 0.040	2.90 × 10^−6^	−0.75 ± 0.41	0.065	1.06 (0.78–1.43)	0.721
6	rs1094818	*WARS2*	1:119238523	G	0.078	-	0.25 ± 0.053	3.49 × 10^−6^	−0.95 ± 0.54	0.080	1.34 (0.91–1.96)	0.140
7	rs78549225	*RBFOX1*	16:6969406	G	0.119	Intron	0.21 ± 0.044	3.50 × 10^−6^	−0.66 ± 0.46	0.148	1.24 (0.88–1.74)	0.222
8	rs78259836	***SNX25***	4:186261649	A	0.105	Intron	0.21 ± 0.046	3.54 × 10^−6^	−1.07 ± 0.47	**0.024**	1.12 (0.79–1.57)	0.522
9	rs7237751	***LOC107984031***	18:47255785	G	0.139	Upstream	0.19 ± 0.041	4.42 × 10^−6^	−0.86 ± 0.42	**0.043**	1.27 (0.93–1.74)	0.137
10	rs12226572	***UBASH3B***	11:122648650	A	0.054	Intron	0.29 ± 0.062	4.54 × 10^−6^	−1.05 ± 0.64	0.100	1.64 (1.07–2.51)	**0.025**
11	rs143090547	*PLPP1*	5:54753484	T	0.056	Intron	0.28 ± 0.062	5.00 × 10^−6^	−1.23 ± 0.63	0.051	1.37 (0.86–2.19)	0.190
12	rs17608925	*ORMDL3*	17:38082831	C	0.066	Intron	0.26 ± 0.056	5.84 × 10^−6^	−0.01 ± 0.58	0.989	1.36 (0.90–2.05)	0.147
13	rs73192989	*RBM19*	12:114580187	T	0.080	-	0.23 ± 0.051	6.77 × 10^−6^	−0.75 ± 0.53	0.155	1.00 (0.67–1.50)	0.989
14	rs199564331	*BRINP3*	1:190127911	D	0.113	Intron	0.20 ± 0.045	9.02 × 10^−6^	−0.56 ± 0.47	0.226	1.27 (0.90–1.80)	0.174
15	rs3773884	*MME*	3:154859650	G	0.052	Intron	0.28 ± 0.063	9.33 × 10^−6^	−0.52 ± 0.64	0.418	1.28 (0.81–2.01)	0.291

SNP, single-nucleotide polymorphism; MAF, minor allele frequency; IDO, indoleamine 2,3-dioxygenase; CKD, chronic kidney disease; eGFR, estimated glomerular filtration rate; β, regression coefficient; S.E., standard error; OR, odds ratio; CI, confidence interval. All analyses were adjusted for age, area, gender, BMI, drinking, smoking, SBP, hs-CRP, and HbA1c. The cutoff *p*-value was *p* < 1 × 10^−5^ for IDO activity and *p* < 0.05 for eGFR and CKD. Genes stated in the manuscript are indicated in bold.

**Table 3:** Rare variants associated with indoleamine 2,3-dioxygenase activity in Koreans.

No.	SNP	Nearest Gene	Chromosome Position	Minor Allele	MAF	HWE *p*-Value	Function	IDO Activity		eGFR		CKD	
								*β* ± S.E	*p*-Value	*β* ± S.E	*p*-Value	OR (95% CI)	*p*-Value
1	rs182145739	*LOC105377444*	4:138651320	T	0.011	1	Intron	0.82 ± 0.13	8.46 × 10^−10^	−1.08 ± 1.36	0.427	2.63 (1.15–6.03)	**0.022**
2	rs149763281	*SLC24A2*	9:20104936	C	0.011	0.277	Intron	0.78 ± 0.13	3.88 × 10^−9^	−1.68 ± 1.36	0.218	0.95 (0.34–2.72)	0.936
3	rs117150322	***TNFRSF19***	13:24120841	A	0.009	0.191	-	0.83 ± 0.15	9.89 × 10^−9^	−3.22 ± 1.49	**0.031**	1.01 (0.30–3.38)	0.994
4	rs188289326	*CACNA2D3*	3:54867936	A	0.010	1	Intron	0.81 ± 0.14	1.02 × 10^−8^	−2.34 ± 1.45	0.107	1.20 (0.49–2.96)	0.690
5	rs146321869	*LOC101928535*	11:106108552	G	0.009	0.183	-	0.83 ± 0.15	1.16 × 10^−8^	−3.34 ± 1.51	**0.026**	1.88 (0.67–5.26)	0.232
6	rs337828	*ARSB*	5:78196735	G	0.009	1	Intron	0.81 ± 0.15	3.55 × 10^−8^	−1.53 ± 1.51	0.310	1.50 (0.55–4.14)	0.431
7	rs58332670	*FSTL5*	4:163207867	C	0.035	0.549	-	0.42 ± 0.08	3.58 × 10^−8^	−2.24 ± 0.79	**4.32 × 10^−3^**	1.43 (0.80–2.57)	0.231

SNP, single nucleotide polymorphism; MAF, minor allele frequency; HWE, Hardy-Weinberg equilibrium; IDO, indoleamine 2,3-dioxygenase; CKD, chronic kidney disease; eGFR, estimated glomerular filtration rate; β, regression coefficient; S.E, standard error; OR, odds ratio; CI, confidence interval. All analyses were adjusted for age, area, gender, BMI, drinking, smoking, SBP, hs-CRP, and HbA1c. Cutoff *p*-values were *p* < 5 × 10^−8^ for IDO activity and *p* < 0.05 for eGFR and CKD. Gene highlighted in the manuscript are indicated in bold.

## Data Availability

The data presented in this study are available on request from the corresponding author. The data are not publicly available due to ethnical concerns.

## Supplementary Materials

The following are available online athttps://www.mdpi.com/article/10.3390/genes12121905/s1, Figure S1: Regional association plots of IDO activity at 10p13, 1p12, 4q35.1, and 18q21.1 loci. The statistical significances between the SNPs near *RSU1* (A), *PDGFD* (B), *SNX25* (C), and *LOC107984031* (D) genes and IDO activity are plotted as −log_10_ *p* values. The purple diamond represents the SNP most strongly associated with IDO activity. Levels of linkage disequilibrium (*r*^2^) of top SNPs and surrounding SNPs are shown in different colors. The regional plots for SNPs were generated via LocusZoom browser (http://locuszoom.org/ (accessed on 16 September 2021)). Figure S2: The KEGG pathway for MAPK signaling pathway. A red box indicates the *PDGFD* gene found in growth factor (GF) according to KEGG. The image of a pathway was generated via KEGG browser (https://www.genome.jp/kegg/ (accessed on 2 September 2021)). Table S1: Rare variants associated with indoleamine 2,3-dioxygenase activity in Koreans.
