# Supplementary material for: Metabolite Genome-Wide Association Study for Indoleamine 2,3-Dioxygenase Activity Associated with Chronic Kidney Disease
Source: Genes (Basel). 2021 Nov 27;12(12):1905. doi: 10.3390/genes12121905 (PMC8701662; doi:10.3390/genes12121905)
Supplement: Supplementary file 1 [file genes-12-01905-s001.zip › Figure S1and 2.pdf]

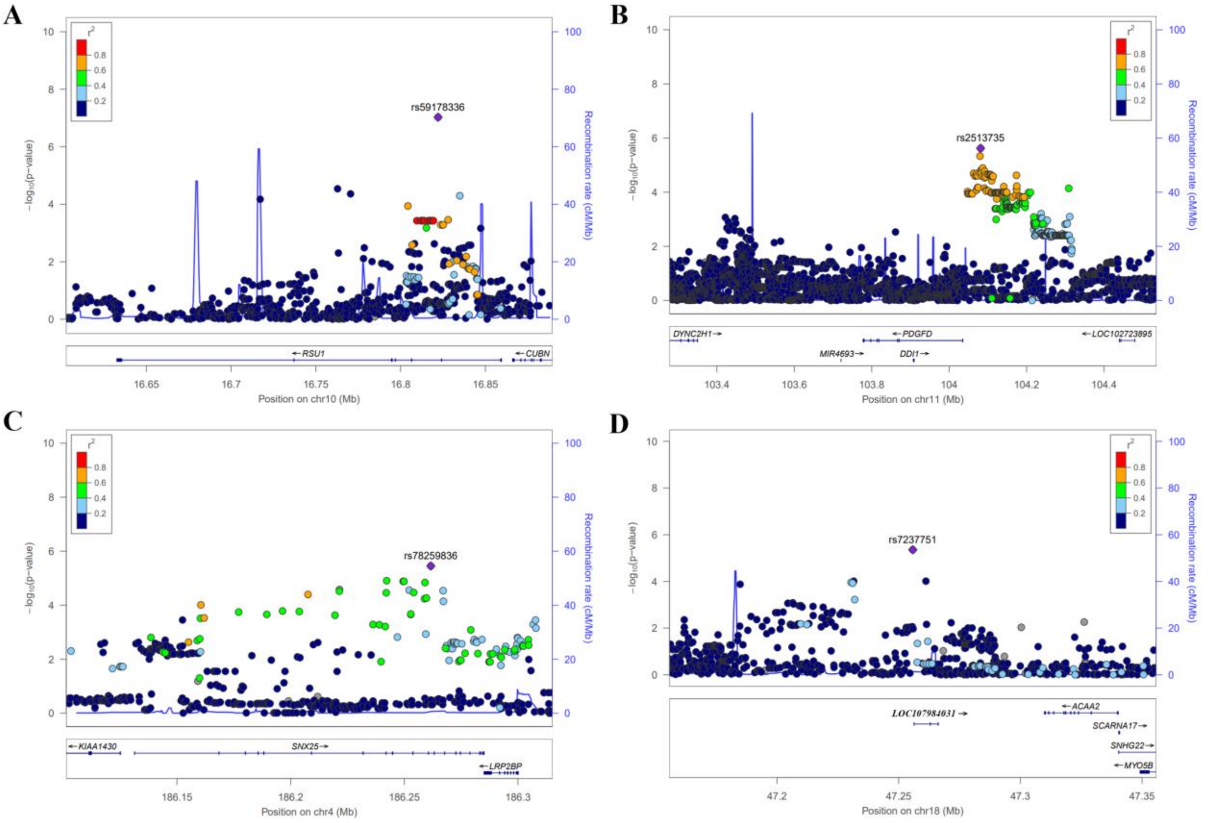

Figure S1: Regional association plots of IDO activity at 10p13, 1p12, 4q35.1, and 18q21.1 loci. The statistical significances between the SNPs near *RSU1* (A), *PDGFD* (B), *SNX25* (C), and *LOC107984031* (D) genes and IDO activity are plotted as $-\log_{10} p$ values. The purple diamond represents the SNP most strongly associated with IDO activity. Levels of linkage disequilibrium (r_2) of top SNPs and sur-rounding SNPs are shown in different colors. The regional plots for SNPs were generated via Lo-cusZoom browser (http://locuszoom.org/ (accessed on 16 September 2021)).

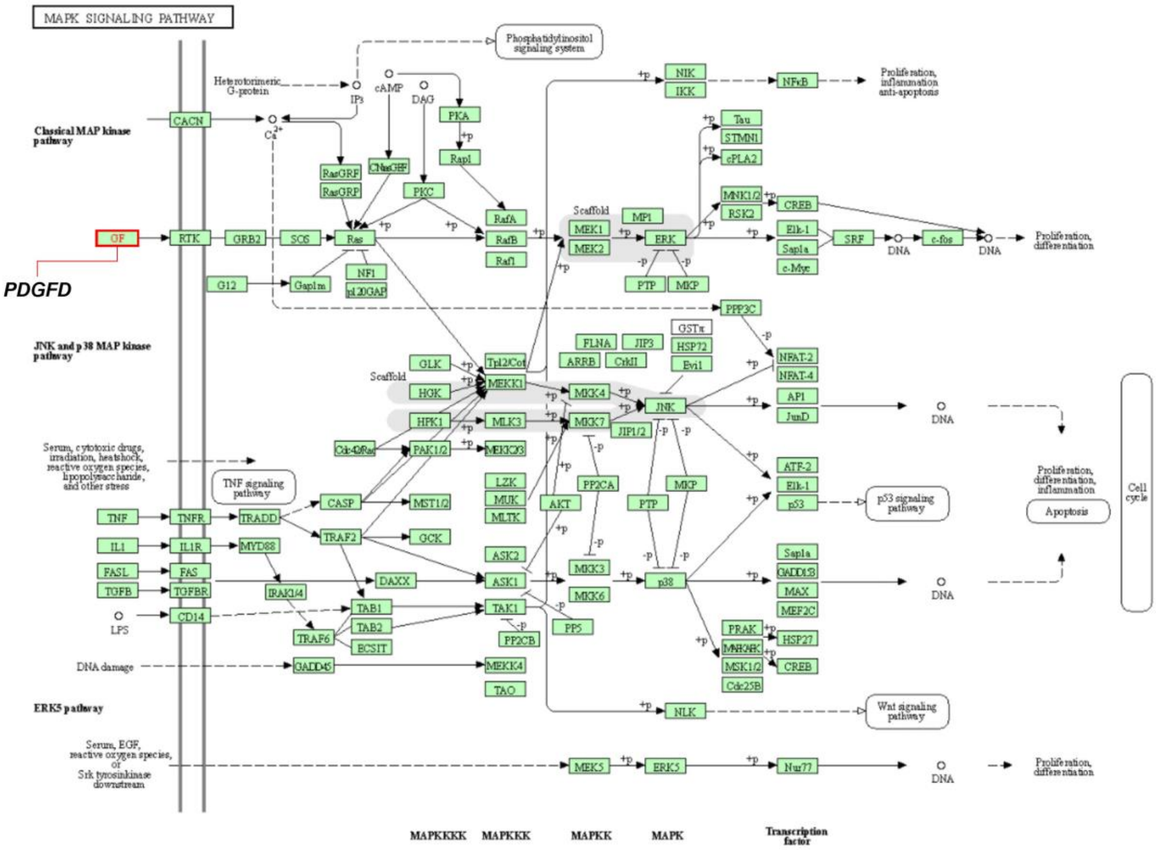

Figure S2: The KEGG pathway for MAPK signaling pathway. A red box indicates the *PDGFD* gene found in growth factor (GF) according to KEGG. The image of a pathway was generated via KEGG browser (https://www.genome.jp/kegg/ (accessed on 2 September 2021)).
